# Enlargements of the Liver.—X

**Published:** 1910-03-12

**Authors:** 


### Medicine.

### ENLARGEMENTS OF THE LIVER.—X.

### ABSCESSES OF THE LIVER (*Concluded.*)

Multiple Abscesses.

The liver is as a rule uniformly enlarged, though post-mortem numerous small abscesses are found scattered throughout its substance. It seldom happens that any of them are large enough and at the same time sufficiently superficial to project from its surface. The condition may be part of a general pyæmia. It is most frequently the result of suppurative pylephlebitis; this is most commonly due to appendicitis. There are three distinct channels by which the micro-organisms can gain access to the liver: the portal veins (portal pyæmia, or suppurative pylephlebitis), the bile ducts (suppurative cholangitis), and the hepatic artery (systemic pyæmia).

The edge of the liver may be felt below the costal margin, and in some cases it may reach down to the level of the umbilicus. The surface may be smooth or nodular, the latter condition depending entirely on the size and the position of the abscesses. When nodules can be detected they are usually felt to be softer than similar nodules would be in a carcinomatous or in a hob-nailed cirrhotic liver. The enlarged organ is usually both tender and painful. Jaundice is more often absent than present in suppurative pylephlebitis, more often present than absent in suppurative cholangitis. Rigors are the rule in both cases. There may be a history of some predisposing cause, such as appendicitis or gall-stones. There is a hectic type of temperature, the pulse is rapid, the tongue tends to be dry and furred, vomiting, and either diarrhœa or constipation occur, and there is steadily progressive emaciation and prostration, ending in death from inanition or cholæmia.

Actinomycosis.

Uncommon though actinomycosis may seem to be, it yet merits attention. The liver may be attacked, either primarily, or more often secondarily to infection of the cæcum or appendix vermiformis. A large single abscess may arise with induration of the surrounding parts, or if the infection has been caused viâ the portal veins, and in addition to actinomyces there are other pyogenetic organisms also present, the abscesses may be multiple. The liver may become enlarged and tender, and the symptoms are those of a chronic pyæmia.

There is a liability in this disease for the affected part of the liver to become adherent to the adjacent parietal peritoneum leading to redness, œdema, and the formation of multiple fistulæ of the abdominal or thoracic wall. A correct diagnosis can only be made by a careful examination of the pus, unless the disease has been recognised already in some other part of the body, such as the tongue, buccal mucosa, pharynx, neck, lung, or appendix vermiformis. The pus may be found to contain yellowish or orange-coloured granules which are about half a millimetre in diameter, and on microscopical examination are found to consist of cocci amidst a mass of mycelial threads whose club-shaped extremities are arranged in the form of rays all round each little mass of the fungus.

Gumma.

Gummata of the liver may be single or multiple, and vary in size from that of a pea to that of a billiard ball or more. They show some predilection for the junction between the right and left lobes, but may occur in the centre of the organ or project from any part of its surface. On section they are usually either greyish white or yellowish and cheese-like; the central part may caseate or even soften and liquefy. The liver may become much enlarged, irregular, and nodular. Where gummata have healed, a depressed fibrous cicatrix results.

A large gumma may give rise to a well-marked localised tumour of the liver, which becomes most prominent if it is situated in the anterior part of the left lobe, where it causes a bulging in the epigastrium. It may be rounded, hard, or soft, and tender. As a result of local perihepatitis, a friction-rub may be heard over it.

There may be no distinctive symptoms or pain, and the most that is complained of may be a sense of weight and fulness in the right hypochondrium. In a few cases pyrexia of the hectic type occurs, so that abscess may be simulated. Evidence of previous syphilis, or the presence of other tertiary syphilitic lesions would point to gumma. If there is any doubt, the result of the administration of potassium iodide may clear up the diagnosis, with the proviso that the non-disappearance of the mass when iodides are given is not proof that it is not gummatous.

General Perihepatitis.

In this affection the liver is usually smaller than normal; occasionally, however, it is enlarged. The capsule in a typical case is enormously thickened and opaque, presenting a curious pitted appearance, so that the surface of the organ resembles to some extent the skin when it has been deeply scarred by smallpox. The edge is either rounded or curled up. The condition is often associated with similar thickening of the whole of the capsule of the spleen, and with chronic peritonitis and ascites. In a large proportion of cases—19 out of 22 in Dr. Hale White's series—it is also associated with granular kidneys.

The most prominent symptom is ascites, which usually requires a large number of tappings; a characteristic of the disease is the rapidity with which the fluid reaccumulates after paracentesis abdominis. Jaundice is very unusual. The liver is not as a rule palpable, but when it is the edge is found to be hard and much rounded and curled up. The latter condition, if it can be made out, is diagnostic.

### Cystic Liver.

This is a very rare condition. It is usually associated with polycystic disease of the kidney, and in all the cases collected by Dr. Hale White there was also cystic disease of the brain. A diagnosis of cystic liver might possibly be made if the liver were obviously enlarged and the kidneys could also be felt to be nodular and big in a young person whose urine was abundant, pale, of low specific gravity, and albuminous.

### Hydatid Cyst.

This disease affects the liver more often than it does any other organ. The size of the cyst may vary from that of a pea to that of a football. The cyst may be embedded in the substance of the liver or it may project from its surface. The physical signs vary according to the position of the cyst.

A small cyst in the centre of the organ may exist without causing either symptoms or physical signs. If it arises from the anterior surface of the left lobe it will produce a rounded prominence in the epigastrium which may be seen to move downwards under the abdominal wall on respiration. Pulsation may be noticed and be determined on palpation to be transmitted from the aorta. If it arises from the lower part of the anterior surface of the right lobe a rounded prominence may be noticed in the right hypochondrium. In both cases the tumour is firm, tense, smooth, painless, and elastic. Fluctuation may sometimes be detected. More often the cyst is so tense as to feel as hard as a board. If the fingers of the left hand are slightly separated from each other and firmly placed over the tumour, and the middle finger is then sharply percussed, a curious sense of vibration is produced, which is termed the hydatid thrill or fremitus. A large hydatid cyst causing a tumour in the epigastrium may be mistaken for a pancreatic cyst, and *vice versa*. Should any doubt arise the stomach may be inflated by giving a Seidlitz powder in two halves separately; if the stomach is found to be anterior to the tumour the latter is probably pancreatic.

If the cyst arises from the under surface of the liver it may project beneath the edge and simulate an enlargement of the gall-bladder. A curious fact about cysts in this position is the frequency with which they occur in the vicinity of the gall-bladder. It is very difficult, in some cases almost impossible, to distinguish one from the other. The presence of jaundice would be more in favour of gall-bladder than hydatid. A distended gall-bladder is more elongated or pyriform in shape, much more movable, and it usually has a constriction in its upper part close to the edge of the liver, whereas a hydatid cyst is more rounded or globular, and it is as a rule firmly fixed to the liver with which it moves.

If the cyst arises from the outer surface of the right lobe it may cause a local bulging of the lower part of the thorax, with filling out and obliteration of the inter-costal spaces; the costal margin may also be pushed forward and the epigastric angle may be widened if the tumour arises from the anterior surface of the right lobe.

If the cyst arises from the upper surface of the liver and enlarges in this direction the liver may be pushed downward and the edge be felt well below the costal margin in the right nipple line; at the same time the diaphragm is pushed upwards, the lung compressed, and the condition may be mistaken for pleuritic effusion at the right base. In both cases there may be deficient movement, filling out and obliteration of the intercostal spaces, diminution or absence of tactile vocal fremitus, impairment of the percussion note, or dulness, diminution of the vesicular murmur, with faint bronchial breathing at the upper limit of the dulness. In the case of hydatid cyst, however, if the dulness is accurately mapped out it is found to have a distinctly arched outline with convexity upwards.

There may be no symptoms until the tumour attains a considerable size, when the patient complains of a feeling of abdominal tightness and distension, and may be conscious of a tumour which he has noticed to have gradually increased in size. As a rule there is no pain, and the tumour is not tender when palpated. If suppuration occurs it may give rise to all the symptoms and signs of hepatic abscess described in a former article, and it should be remembered that suppurating hydatid cyst is the commonest form of large single abscess in the liver of a patient who has never resided out of Great Britain and Ireland. If the cyst becomes adherent to the stomach or intestines the contents of the cyst may be vomited or passed into the fæces. If the cyst ruptures into the peritoneal cavity acute pain results, and a profuse rash may be produced all over the body. If the cyst compresses the inferior vena cava it may produce œdema of the legs; if the common bile duct, jaundice; if the portal vein, ascites. Pulmonary embolism even has resulted from a cyst opening into the inferior vena cava.

The chief evidence then of an enlargement of the liver being due to hydatid disease is the presence of a considerable, rounded, tense, elastic, cyst-like tumour, which is not tender and which does not give rise to pain or other severe constitutional disturbances unless some such complication as rupture or suppuration has supervened. Should there be any doubt an exploratory laparotomy should be made.

*Hydatid fluid* is colourless, clear, or faintly opalescent, alkaline in reaction, and of specific gravity 1,006 to 1,010. It contains no albumen. It may or may not contain bile—a living cyst does not become bile stained, a dead one does. Traces of grape sugar or other substance capable of reducing Fehling's solution, a considerable amount of sodium chloride, and traces of succinic acid may be present. If the fluid is shaken a number of minute white specks may often be seen floating in it. On microscopical examination scolices may be found—round or oval bodies with a slight constriction at one end with a crown of hooklets and four suckers. Sometimes, however, the cyst is barren, containing no scolices. An examination of the centrifugalised deposit may lead to the detection of detached hooklets and shreds of laminated membrane with transverse striation and a granular inner surface. The hooklets are pathognomonic.

